# Custom-Made Versus Non-Custom Mouthguards in the Prevention of Sports-Related Oro-Dento-Facial Trauma: A Scoping Review of the Clinical Evidence

**DOI:** 10.3390/bioengineering13080916

**Published:** 2026-08-13

**Authors:** Giulia Perrotta, Alessio Verdecchia, Renisa Basha, Barbara Frenna, Laura Carboni, Giulia Attene, Simone Parrini, Gabriele Rossini, Claudia Dettori, Enrico Spinas

**Affiliations:** 1 Department of Surgical Sciences, Postgraduate School in Orthodontics, University of Cagliari, 09124 Cagliari, Italy; drgiuliaperrotta@gmail.com (G.P.); renisabasha@gmail.com (R.B.); barbarafre@outlook.it (B.F.); lrcarbons@gmail.com (L.C.); giuliaattene@yahoo.it (G.A.); enricospinas@tiscal.it (E.S.); 2 Orthodontics Division, Instituto Asturiano de Odontología, Universidad de Oviedo, 33006 Oviedo, Spain; 3 Department of Orthodontics, University of Siena, 53100 Siena, Italy; 4 Private Practice, 20100 Milan, Italy; dr.gabriele.rossini@gmail.com; 5 Department of Surgical Sciences, School of Dental Medicine, University of Cagliari, 09124 Cagliari, Italy; claudia.dettori@gmail.com

**Keywords:** mouthguard, custom-made mouthguard, sports dentistry, oro-dento-facial trauma, traumatic dental injuries, prevention, ethylene-vinyl acetate, scoping review

## Abstract

Sports-related oro-dento-facial trauma is a major clinical and socioeconomic burden, with oro-dento-facial injuries affecting approximately 30–40% of athletes. Mouthguards are widely recommended as primary protective devices, yet the comparative effectiveness of custom-made versus stock and boil-and-bite designs remains unclear. This scoping review aimed to map the clinical evidence on different mouthguard types for preventing sports-related oro-dento-facial injuries. The review followed the PRISMA-ScR guidelines. PubMed, Embase, Scopus, Web of Science, and the Cochrane Library were searched on 22 January 2026, without restrictions on language, date, or design. Eligibility was defined using the PICOS framework, and three reviewers independently performed selection and data extraction (Cohen’s kappa = 0.79). Of 820 records, eight studies published between 1989 and 2025 met the inclusion criteria, covering contact and non-contact sports across a wide age range. Custom-made mouthguards, mainly fabricated from ethylene-vinyl acetate, were consistently associated with reductions in dental trauma, intraoral lacerations, and temporomandibular joint injuries, with statistically significant effects in several studies (*p* < 0.05 to *p* < 0.001). Heterogeneity in design, materials, and outcomes precluded quantitative synthesis. Custom-made mouthguards are associated with favourable clinical outcomes; however, direct comparative evidence remains limited, and the heterogeneity of the available studies precludes definitive conclusions regarding their superiority over non-custom devices. Standardised reporting and large prospective studies are needed to define optimal material and design parameters.

## 1. Introduction

Oro-dento-facial injuries represent a substantial clinical and socioeconomic burden in both contact and non-contact sports, frequently involving soft tissues, dental structures, and the facial skeleton, with a reported prevalence of approximately 30–40% [1,2,3]. Sports-related facial trauma contributes substantially to maxillofacial fractures, with patterns varying according to the type of sport and geographical context. The nasal bones, zygomatic complex, and mandible are the most frequently affected sites, particularly in contact or high-velocity disciplines. Although the incidence is generally higher in younger individuals and in males, exposure-adjusted data indicate smaller gender-related differences than those previously reported [2].

Within this heterogeneous spectrum of orofacial trauma, traumatic dental injuries (TDIs) represent a particularly relevant subgroup because of their high frequency, potential long-term sequelae, and preventability. In the general population, TDIs are estimated to affect approximately 15.2% of permanent teeth and 22.7% of primary teeth worldwide, with more than one billion individuals having experienced a dental trauma [4]. Participation in sports further increases this burden, especially in contact, collision, and combat disciplines. Meta-analytic evidence indicates that dental injuries represent the most frequent injury type and affect approximately 20–30% of athletes [3,4,5]. These findings underscore the influence of sport type, age, exposure level, protective-device use, and study methodology on reported prevalence estimates.

Despite this, awareness and use of preventive devices remain inadequate: in paediatric populations, only approximately 10% of young athletes were reported to be aware of mouthguards, while consistent use was observed in approximately 1% [6].

The Andreasen classification of dental trauma [7], subsequently adopted by the International Association of Dental Traumatology (IADT) and updated in 2020, distinguishes injuries affecting hard dental tissues and the pulp from those involving the periodontal and dento-alveolar structures. Intrusive and lateral luxations are among the most severe paediatric injuries; their management is complex, and the prognosis depends on the degree of root maturity and the severity of the trauma, although orthodontic repositioning may represent a valid conservative therapeutic option [8]. The IADT guidelines provide a robust framework to support clinical decision-making, considering the timing of intervention, the age of the patient, and the severity of the injury [9,10,11].

Risk factors for TDIs can be classified as intrinsic and extrinsic. Intrinsic factors include Angle Class II Division 1 malocclusion, lip incompetence, incisor proclination, and increased overjet. The maxillary central incisors are the teeth most frequently affected, accounting for 60–80% of cases, followed by the maxillary lateral incisors [12,13]. Extrinsic factors are mainly related to sports and domestic accidents. Higher-risk sports include squash, wrestling, equestrian disciplines, rugby, handball, basketball, and football, with injury patterns influenced by equipment, impact velocity, level of physical contact, and number of participants [14]. Sports-related trauma has been reported to account for 10–39% of all dental injuries in children, although estimates vary according to age, sport type, and study setting. Therefore, primary prevention represents a clinical priority in young athletes [15]. Severe traumatic dental injuries, such as avulsion of permanent teeth, may require complex emergency management and prolonged follow-up, with long-term outcomes often remaining unpredictable because of complications such as ankylosis and root resorption. These clinical, functional, aesthetic, and psychological consequences make primary prevention a major priority in children and adolescent athletes [16].

These risks justify the need—and, in some contexts, the obligation—to use protective devices such as helmets and mouthguards. Mouthguards provide direct prevention by protecting the maxillary incisors, reducing intraoral soft-tissue lacerations, and limiting indirect injuries caused by sudden occlusal contact. Beyond oral and dentoalveolar injuries, sports-related concussions represent a major public health concern, particularly among adolescents, with sports accounting for a substantial proportion of concussion cases in individuals aged 11–19 years. Although the protective role of mouthguards against concussion remains less conclusive than their established effect on dental and intraoral trauma, their potential ability to attenuate mandibular impact forces may support their inclusion within broader sports injury-prevention strategies [17,18]. Promoting mouthguard use among young athletes is essential, as severe dental injuries frequently occur during the growth period, particularly between 8 and 10 years of age. Early introduction may improve long-term acceptance and compliance [19], with younger athletes showing greater adherence than those who have been accustomed to practising without protection [20,21].

Despite their well-documented benefits, mouthguards are often avoided because of discomfort, poor retention, and difficulties with breathing or phonation, frequently attributable to inadequate materials and non-customised designs. An optimal mouthguard should fulfil specific clinical and technical requirements [6]: it should be resilient and impact-absorbing, hygienic, saliva-resistant, stable when out of occlusion, and should neither interfere with occlusion nor produce unintended orthodontic effects.

Mouthguards are conventionally classified as stock, semi-custom (boil-and-bite), and custom-made devices [6,22,23]. Stock devices typically provide poor fit and retention; boil-and-bite models offer moderate, inexpensive protection but may fit inadequately; custom-made mouthguards provide superior protection, comfort, and functional adaptation. Device selection should be informed by sport-specific risk, motor patterns, and biomechanical demands.

Unlike previous systematic reviews and umbrella reviews, which have primarily examined the overall protective effect of mouthguard use, the present scoping review maps how different mouthguard types have been investigated and reported across the clinical literature. It examines the distribution of evidence according to sport, study design, comparator, device characteristics, and injury outcome, while distinguishing direct comparisons between custom-made and non-custom mouthguards from indirect comparisons between mouthguard users and non-users. This approach also allows the identification of reporting and methodological gaps that currently limit conclusions regarding comparative effectiveness.

On this basis, the present scoping review aims to map the existing scientific evidence on the use of different types of mouthguards in sports, with particular attention to the preventive effect of custom-made mouthguards, compared with stock and self-adapted devices, on sports-related injuries involving the lower third of the face. These include dental and dentoalveolar trauma, intraoral soft-tissue injuries, maxillary and mandibular bone injuries, and temporomandibular joint injuries.

## 2. Materials and Methods

### 2.1. Information Sources, Protocol, and Search Strategy

This scoping review was conducted in strict accordance with the Preferred Reporting Items for Systematic Reviews and Meta-Analyses extension for Scoping Reviews (PRISMA-ScR) guidelines [24]. The study protocol was defined a priori and registered on the Open Science Framework (OSF; registration DOI: [10.17605/OSF.IO/KVZPU]) to ensure a systematic, transparent, and reproducible approach to the available literature.

The primary research question underpinning this review was “How are custom-made mouthguards investigated and reported in comparison with semi-custom (boil-and-bite) and stock mouthguards in relation to the prevention of sports-related orofacial trauma?” A secondary objective was to identify the specific types of orofacial injuries (e.g., bone fractures, dental trauma, soft-tissue lacerations, and temporomandibular joint [TMJ] injuries) reported across different mouthguard investigations.

A comprehensive and systematic literature search was performed on 22 January 2026 in the following electronic databases: PubMed, Embase, Scopus, Web of Science, and the Cochrane Library. To maximise coverage, a manual search of the reference lists of all included studies and of relevant previous reviews was also performed to identify any further eligible publications. No restrictions were applied regarding publication date, language, or study design in order to minimise selection bias.

Although the review focused on prevention, the term “treatment” was retained as a supplementary search term to maximise search sensitivity and capture potentially relevant studies indexed under broader trauma-management terminology. Eligibility screening was nevertheless restricted to studies reporting preventive outcomes associated with mouthguard use.

The detailed search strategies for each database are presented in Table 1.

### 2.2. Eligibility Criteria

The PICOS framework (Population, Intervention, Comparison, Outcomes, Study design) was used to define eligibility as follows:

Population (P): athletes of all ages and sexes, without physical or mental disabilities;

Intervention (I): use of sports-specific custom-made mouthguards;

Comparison (C): use of mouthguards of a different type compared with the sample group;

Outcome (O): effectiveness of custom-made mouthguards in sports traumatology;

Study Design (S): experimental, observational, and comparative studies.

Eligible studies were those assessing the effectiveness of different types of mouthguards in the prevention of sports-related orofacial injuries and their recurrences. Studies were excluded if they did not specify the type of mouthguard used either in the sample or in the control group or if the population was not engaged in sports activities. The detailed inclusion and exclusion criteria are reported in Table 2.

### 2.3. Data Extraction and Synthesis

Three reviewers (G.P., R.B., B.F.) independently conducted the literature search and screened the studies for eligibility. Inter-reviewer agreement was assessed using Cohen’s kappa coefficient, which indicated substantial agreement (κ = 0.79) [25]. Discrepancies were resolved through discussion with a fourth reviewer (E.S.).

For each included study, the following data were systematically extracted into a standardised data-extraction form:

**Study characteristics**: authors, year of publication, country, and study design;

**Sample data**: size, age, sex distribution, specific sport practised, and type of comparison;

**Intervention details**: type of mouthguard (custom-made, semi-custom, or stock), material, and thickness;

**Clinical outcomes**: intraoral/extraoral soft-tissue injuries, dental trauma, bone injuries, and TMJ injuries.

For each clinical outcome, statistically significant values were highlighted, together with clinically relevant but non-statistically significant findings, unreported values, values reported only descriptively rather than analytically, and values for which the type of mouthguard associated with the outcome was not specified.

## 3. Results

### 3.1. Study Selection

The initial systematic search across the electronic databases identified a total of 820 records: PubMed (*n* = 323), Web of Science (*n* = 215), Embase (*n* = 229), Scopus (*n* = 11), and the Cochrane Library (*n* = 38). This was supplemented by 4 additional records identified through the manual screening of reference lists. After the removal of 362 duplicate citations, 458 unique records remained for screening. In the first phase of selection, 441 records were excluded following a preliminary screening of titles and abstracts, as they did not align with the scope of the study.

**Figure 1:**
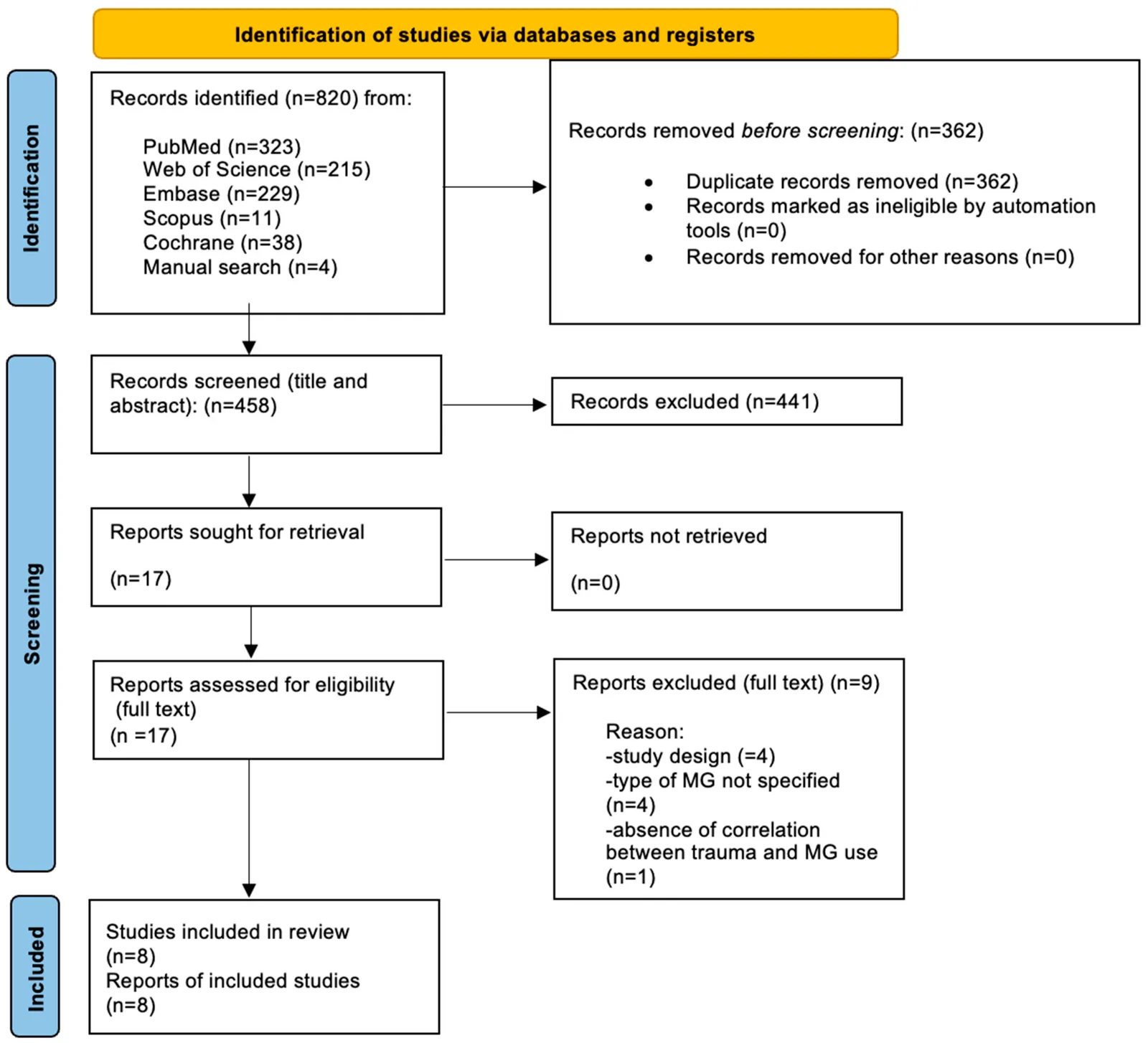
PRISMA 2020 flow diagram of the study selection process.

The remaining 17 records were sought for retrieval. Of these 17 full-text articles assessed for eligibility, 9 were excluded due to a lack of specificity regarding mouthguard typology, the inclusion of non-athletic populations, or the absence of a clear correlation between mouthguard use and orofacial trauma. Ultimately, 8 studies fulfilled all eligibility requirements and were included in the qualitative synthesis. The selection process is summarised in the PRISMA 2020 flow diagram (Figure 1).

### 3.2. General Characteristics of the Included Studies

The general characteristics of the included studies are summarised in Table 3. The studies were published between 1989 and 2025 and were conducted across different geographical regions, including North America, Asia, Europe, and Oceania. Specifically, the research was carried out in the United States [26,27], Japan [28], Canada [29], Australia [30], Switzerland [31], and India [32,33].

The included studies investigated a wide range of sports, predominantly contact or high-risk disciplines such as American football, rugby, and combat sports [26,28,29], as well as team sports and activities with variable traumatic exposure, including basketball, soccer, handball, and roller skating [27,33].

The methodological designs were heterogeneous and included cross-sectional observational studies [26,28,31], prospective observational or cohort studies [27,32], randomised controlled trials [29,30], and one non-randomised interventional study [33]. Sample sizes ranged from small cohorts to large population-based studies involving several thousand athletes.

The study populations spanned a broad demographic spectrum, including children and adolescents (aged 8–18 years) as well as adult athletes at both university and professional levels.

**Figure 2:**
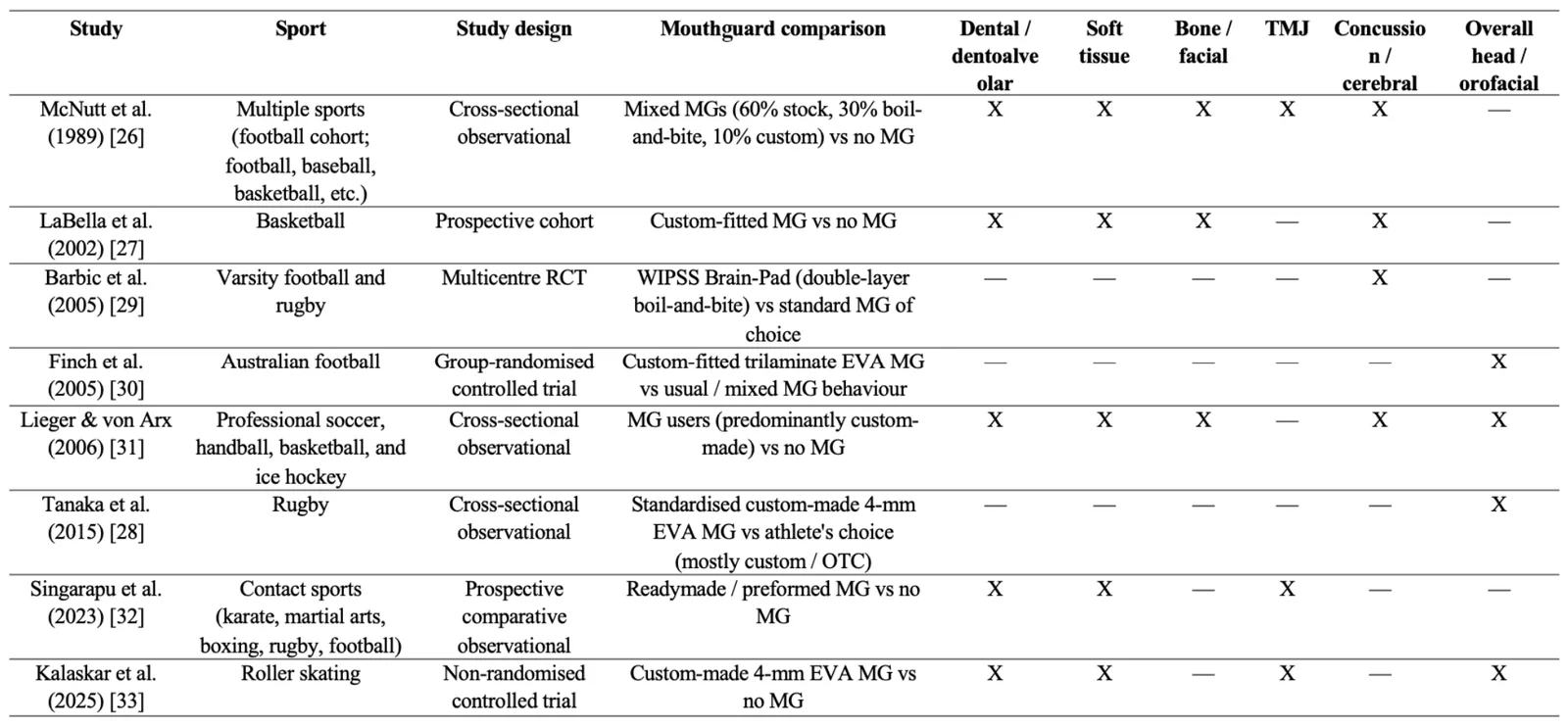
Evidence map of the included studies according to sport, study design, mouthguard type, comparator, and assessed clinical outcomes [26,27,28,29,30,31,32,33].

Regarding the preventive interventions, the included studies evaluated the comparative efficacy of stock, mouth-formed (boil-and-bite), and custom-made mouthguards, often within the same study population [26]. When reported, custom-made mouthguards were predominantly fabricated from ethylene-vinyl acetate (EVA), with occasional multilaminate designs or a standardised thickness [28,30,33]. Comparison groups typically consisted of athletes using non-customised devices or no mouthguard at all during athletic activity. However, not all studies clearly classified the two groups as sample and control groups; therefore, these are reported as Group 1 and Group 2 in Table 4. Figure 2 provides an evidence map of the included studies according to sport, study design, mouthguard type, comparator, and assessed clinical outcomes, offering an overview of the methodological and clinical characteristics of the available evidence.

### 3.3. Structural Characteristics and Materials of the Analysed Mouthguards

The analysis of the included studies reveals marked heterogeneity in the types of mouthguards evaluated and in the materials used for their fabrication, as detailed in Table 5. The preventive interventions primarily focused on comparing custom-fitted (or custom-made) mouthguards with standardised alternatives, such as stock or mouth-formed (boil-and-bite) devices.

In most investigations, one group used professionally fabricated custom devices, while the other group consisted of athletes wearing either non-customised devices or no protection at all. About material composition, ethylene-vinyl acetate (EVA) was the predominant material reported for custom-made devices. Notably, specific fabrication techniques were described in the more recent or specialised studies: Finch et al. (2005) [30] used a trilaminate EVA design, while Tanaka et al. (2015) [28] and Kalaskar et al. (2025) [33] specified a standardised thickness of 4 mm for Group 1.

In contrast, a considerable proportion of the earlier literature—such as McNutt et al. (1989) [26]—did not specify the exact material or thickness used, simply categorising the devices by their general type. The shift towards more detailed reporting in later studies, including the description of double-layer boil-and-bite designs such as the WIPSS Brain-Pad [29], reflects an increasing scientific focus on the biomechanical properties of these protective devices.

### 3.4. Clinical Outcomes and Preventive Efficacy

The clinical impact of mouthguard use across the included studies demonstrates an overall reduction in orofacial injuries, encompassing dental, skeletal, and soft-tissue trauma, as summarised in Table 6.

The evidence on dental trauma consistently indicates that both standardised and custom-fitted mouthguards significantly reduce the incidence of fractures and luxations [26,27]. In particular, studies conducted in professional cohorts reported a statistically significant decrease in dental injuries (*p* < 0.05), underscoring the role of mouthguards as a primary protective mechanism against the high prevalence of TDIs. Reductions in dental trauma were also reported by Lieger and von Arx (2006) [31], Singarapu et al. (2023) [32], and Kalaskar et al. (2025) [33], although no inferential statistical analysis was provided.

The protective effect of these devices also extends to soft-tissue preservation, with both intraoral and extraoral regions being effectively shielded. Significant reductions in intraoral lacerations were reported across multiple athletic disciplines [26,33]. In addition, custom-fitted models were associated with superior outcomes, whereas extraoral soft-tissue injuries were reported to be reduced with mouthguard use, although the specific mouthguard type was not specified [31].

Furthermore, the available data suggest a broader protective effect, particularly with regard to bone injuries and the TMJ. Several authors documented a reduction in bone injuries [26,31,33], with some interventions reporting a high level of statistical significance (*p* < 0.001) for TMJ protection [32,33]. Overall, these findings indicate that precision-fitted mouthguards optimise force dissipation, thereby reducing the risk of complex orofacial fractures and joint trauma in both contact and high-velocity sports.

Finch et al. (2005) [30] and Tanaka et al. (2015) [28] did not distinguish among different injury types; nevertheless, the reduction in orofacial injuries associated with the use of custom-made mouthguards was statistically significant.

## 4. Discussion

### 4.1. Main Findings

The role of mouthguards in preventing dentofacial injuries in sports is now widely recognised in the scientific literature. Numerous studies have demonstrated that mouthguards significantly reduce the frequency and severity of orofacial injuries in contact sports. Athletes who do not wear mouthguards have been reported to have a 1.6 to 1.9 times higher risk of injury compared with users [17,34].

This scoping review confirms that orofacial injuries represent a significant clinical and socioeconomic burden in sports, with a prevalence of approximately 40% among athletes. McNutt et al. (1989) [26] reported that 75% of the recorded oral injuries occurred while athletes were not wearing a mouthguard, supporting the general preventive value of mouthguard use. However, McNutt et al. evaluated a heterogeneous group of mouthguard users, predominantly using stock and mouth-formed devices, and did not stratify outcomes by mouthguard type. Therefore, this study supports the general protective effect of mouthguard use but cannot inform comparisons between custom-made and non-custom devices.

The incidence of trauma varies according to the sport: Lieger and von Arx (2006) [31] reported dental injuries in 59% of unprotected ice hockey players, compared with 24% in soccer players, highlighting the influence of contact intensity and sport-specific dynamics. Spinas et al. (2014) [35] further demonstrated that, even in medium-risk sports such as basketball, the discontinuation of protective devices was associated with severe injuries, including dental dislocations requiring endodontic treatment. The available evidence supports a protective effect of mouthguards against sports-related orofacial injuries, particularly dental trauma and intraoral soft-tissue injuries. Custom-fitted or custom-made mouthguards were associated with reductions in intraoral soft-tissue injuries, dental trauma, and TMJ injuries, with statistically significant effects reported for dental trauma by LaBella et al. (2002) [27] and for TMJ injuries by Singarapu et al. (2023) [32] and Kalaskar et al. (2025) [33]. Nevertheless, the findings remain heterogeneous, with several studies reporting only descriptive or non-significant reductions. Evidence regarding extraoral soft-tissue and bone injuries is limited, although reductions were observed with custom-fitted devices. Overall, these data support the preventive role of custom-made mouthguards and underscore the need for standardised studies across injury categories.

Importantly, several included studies compared mouthguard users with non-users rather than directly comparing custom-made and non-custom devices. Therefore, these studies primarily support the general protective effect of mouthguard use, whereas evidence regarding the specific advantage of customisation remains more limited. Consequently, the apparent superiority of custom-made devices should be interpreted cautiously and reflects the overall direction of the available evidence rather than consistent direct head-to-head comparisons.

### 4.2. Comparison with Previous Reviews and Technical Recommendations

The findings of the present scoping review are generally consistent with previous systematic reviews, umbrella reviews, and consensus recommendations supporting the protective role of mouthguards against dentoalveolar injuries in sports. In a recent umbrella review, Agarwal et al. (2025) [34] showed that mouthguard use significantly reduces dentofacial injuries, particularly tooth fractures and avulsions, thereby confirming their protective role in athletes. Longitudinal clinical evidence further substantiates this: Spinas et al. (2018) [21] found that athletes using mouthguards following trauma experienced no further complications or re-injuries over a five-year follow-up, validating post-traumatic preventive protocols. However, the level of protection differs markedly according to device type. The available evidence suggests that custom-made mouthguards may provide greater protection than stock or boil-and-bite devices, mainly because of improved impact-force distribution and greater intraoral stability. Spinas and Savasta (2007) [6] emphasised the role of mouthguards in preventing sports-related TDIs, while later consensus recommendations suggested a minimum thickness of 3 mm on the labial and occlusal surfaces and 2 mm on the palatal surface [36,37]. Conversely, boil-and-bite mouthguards often undergo uneven thinning during intraoral moulding, which reduces their capacity for impact dissipation. EVA remains the most widely cited and studied material in the literature [28,30,33].

### 4.3. Clinical Implications, Compliance, and Individual Risk Factors

Beyond sport-related determinants, individual susceptibility also plays an important role in the occurrence of dental trauma. Specific dentoskeletal characteristics, including Angle Class II Division 1 malocclusion, lip incompetence, and increased overjet, expose the maxillary incisors to a higher risk of traumatic injury [16]. In high-risk individuals, custom-made mouthguards may offer an additional protective benefit by compensating for reduced natural lip protection. Consistent with this, Mordini et al. (2021) [38] reported that the severity of dental luxation injuries is inversely related to the quality of the mouthguard fit, further emphasising the importance of professionally fabricated devices.

Despite robust evidence supporting their efficacy, mouthguard use among athletes remains suboptimal [39]. Common barriers include discomfort, difficulty breathing, and interference with speech [40], whereas adequate fit and retention are key determinants of compliance, particularly in adolescents [30]. This reflects the need to balance protection, stability, and tolerability: greater mouthguard bulk may enhance intraoral retention and stability, but it can also increase perceived discomfort; conversely, thinner devices or mouthguards with reduced vestibular extension may be more comfortable for athletes, although potentially less stable. In this context, carefully designed custom-made mouthguards may offer a clinically favourable compromise, providing adequate stability and protection while maintaining acceptable comfort, even when a greater material volume is required. Because comfort and wearability strongly influence adherence, custom-made mouthguards may be preferable to self-adapted devices. DeYoung et al. (1994) [41] reported that, although both types provided protection, athletes consistently preferred custom-made devices. Similarly, Spinas et al. (2016) [42] observed good 9-month compliance with custom-made mouthguards in young athletes, although electronic monitoring may have influenced their behaviour. Risk perception also affects adoption: Yamada et al. (1998) [43] found that more than 80% of soccer players considered mouthguards unnecessary despite the well-documented injury risks, suggesting that behavioural and cultural factors contribute substantially to their limited use.

Recent technological advances in digital dentistry and three-dimensional printing have opened new perspectives for mouthguard design and fabrication. These technologies allow greater anatomical precision and customisation, potentially improving comfort, stability, acceptance, and long-term adherence. In parallel, the early introduction of protective devices is considered an important preventive strategy. Matalon et al. (2008) [19] recommended introducing mouthguard use between 8 and 10 years of age, since early adoption may facilitate the development of long-term protective habits in young athletes.

From a clinical perspective, these findings support the promotion of mouthguard use in sports associated with a high risk of dentofacial trauma. The identification of individual risk factors may assist clinicians in recommending targeted preventive strategies. Sports dentistry therefore plays an important role in mouthguard fabrication, athlete counselling, and educational programmes for athletes, coaches, and parents.

### 4.4. Research Gaps and Future Directions

Several important evidence gaps emerged from this review. Direct comparisons between custom-made and non-custom mouthguards remain limited, and the available studies are heterogeneous in design, population, comparator, and outcome assessment. The methodological characteristics of the included evidence was also variable. Several studies were observational or non-randomised, some included relatively small samples, and mouthguard types and outcomes were not always clearly reported or appropriately stratified. These limitations reduce the certainty of the evidence and preclude definitive conclusions regarding comparative effectiveness.

In addition, differences in mouthguard materials, thickness, fabrication techniques, and assessment protocols limit comparability across studies. Consequently, no standardised evaluation protocol is currently available to assess the comparative performance of different mouthguard designs. Moreover, the available literature does not yet allow the identification of an optimal combination of material and thickness that would maximise protection against TDIs while ensuring adequate comfort, wearability, and athlete acceptance, all of which are essential for maintaining high compliance. Future research should focus on large prospective studies involving diverse athletic populations and using standardised protocols to compare custom-made, boil-and-bite, and stock mouthguards, with detailed reporting of device type, material, thickness, and fabrication technique. As TDIs are accidental events that cannot be experimentally induced, their occurrence should be investigated through large prospective cohorts, with systematic recording of athlete exposure, potentially traumatic events, mouthguard use, and actual trauma incidence.

### 4.5. Strengths of This Review

This scoping review has several strengths. First, it provides an updated synthesis of the available clinical evidence on the preventive role of mouthguards in preventing dentofacial injuries in athletes, encompassing randomised and non-randomised trials, cohort studies, and cross-sectional observational studies. Additional evidence from systematic reviews, umbrella reviews, and epidemiological studies was used to support the discussion and to place the results within the broader context of sports dentistry research. Second, the critical appraisal of the literature highlights not only the well-established benefits of mouthguards but also the methodological limitations of existing studies, particularly the lack of standardisation. Finally, this review addresses both the biomechanical and behavioural aspects influencing mouthguard use, offering a broader perspective on injury-prevention strategies in sports.

## 5. Conclusions

This scoping review mapped the available evidence on the effectiveness of mouthguards in preventing sports-related orofacial trauma. Overall, the literature supports the use of mouthguards as a primary preventive strategy to reduce the incidence and severity of dentofacial injuries across a wide range of sports.

The protective effect appears to depend on device quality, intraoral fit, and athlete compliance. The available evidence suggests that custom-made mouthguards may provide greater protection than non-custom devices owing to their superior adaptation, stability and comfort; however, the heterogeneity of the included studies precludes definitive conclusions regarding comparative effectiveness. Educational interventions, the early use of protective devices in young athletes, and the application of digital technologies for individualised fabrication may further improve adherence and preventive effectiveness.

However, the current evidence remains limited by marked heterogeneity in study design, outcome measures, and follow-up protocols. Moreover, key mouthguard features—including material, thickness, fabrication technique, and design—are often poorly reported, reducing comparability between studies and precluding firm conclusions regarding optimal device characteristics.

The main evidence gaps include the limited number of direct comparative clinical studies, heterogeneous study designs and outcome definitions; incomplete reporting of mouthguard material, thickness, and fabrication technique; and the lack of large prospective studies directly comparing different mouthguard types.

Future studies should adopt standardised reporting of mouthguard properties and clinical outcomes and should include large, high-quality prospective cohorts in athletic populations. Such data are needed to clarify the relative effectiveness of different mouthguard types and to define the optimal balance between protection, comfort, and compliance. Within these limitations, the routine use of custom-made mouthguards should be considered a key component of contemporary sports dentistry, with the aim of reducing preventable orofacial injuries in athletes.

## Figures and Tables

**Table 1:** Search strategy for each database.

Database	Search Strategy
PubMed	((“prevention”[All Fields] AND “treatment”[All Fields] AND “dental trauma”[All Fields] AND “sports-related dental trauma”[All Fields]) OR (“mouth protectors”[MeSH Terms] OR (“mouth”[All Fields] AND “protectors”[All Fields]) OR “mouth protectors”[All Fields] OR “mouthguard”[All Fields] OR “mouthguards”[All Fields])) AND “athletes”[All Fields]
Web of Science	“prevention” (All Fields) AND “treatment” (All Fields) AND “dental trauma” (All Fields) AND “sport-related dental trauma” (All Fields) OR “mouthguard” (All Fields) AND “athletes” (All Fields)
Cochrane Library	“prevention” in Title Abstract Keyword AND “treatment” in Title Abstract Keyword AND “sports-related dental trauma” in Title Abstract Keyword OR “mouthguards” in Title Abstract Keyword AND “athletes” in Title Abstract Keyword
Scopus	(TITLE-ABS-KEY (“prevention”) AND TITLE-ABS-KEY (“treatment”) AND TITLE-ABS-KEY (“dental trauma”) AND TITLE-ABS-KEY (“sports-related dental trauma”) OR TITLE-ABS-KEY (“mouthguards”) AND TITLE-ABS-KEY (“athletes”))
Embase	((‘prevention’/exp OR ‘prevention’) AND ‘treatment’ AND ‘dental trauma’ AND ‘sports-related dental trauma’ OR mouthguards) AND ‘athletes’

**Table 2:** Inclusion and exclusion criteria.

Inclusion criteria	Studies specifying the type of mouthguard used. Studies including participants engaged in sports activities. Studies evaluating the relationship between sports-related orofacial trauma and mouthguard use.
Exclusion criteria	In vitro and animal studies. Case reports, case series, and review articles. Studies not specifying the type of mouthguard used. Studies lacking a clear association with sports activity or trauma-related outcomes.

**Table 3:** General characteristics of the included studies.

No.	Authors (Year)	Country	Sport	Study Design	Group 1 (Age, Sex)	Group 2 (Age, Sex)
1	McNutt et al. (1989) [26]	USA	Football, baseball, basketball	Cross-sectional observational	Mean age 15.5 y; M	Age range 10–18 y; M
2	LaBella et al. (2002) [27]	USA	Basketball	Prospective cohort	Mean age: NR; M	Mean age: NR; M
3	Barbic et al. (2005) [29]	Canada	Varsity football and rugby	RCT	Mean age 20.8 ± 2.1 y; M (football 394, rugby 129), F (rugby 123)	Mean age 20.9 ± 1.9 y; M (football 394, rugby 129), F (rugby 123)
4	Finch et al. (2005) [30]	Australia	Football	RCT	Seniors 24 ± 5.1 y; Juniors (U16–18) 17 ± 1.2 y; M	Seniors 26 ± 4.6 y; Juniors (U16–18) 16 ± 1.1 y; M
5	Lieger & von Arx (2006) [31]	Switzerland	Soccer, handball, basketball, ice hockey (professional)	Cross-sectional observational	Mean age 26 y; M	Mean age 26 y; M
6	Tanaka et al. (2015) [28]	Japan	Rugby	Cross-sectional observational	Mean age 17.0 ± 0.7 y; M	Mean age 22.5 ± 2.6 y; M
7	Singarapu et al. (2023) [32]	India	Contact sports (karate, martial arts, boxing, rugby, football)	Prospective observational (comparative)	Mean age 25.5 y; M/F	Mean age 25.5 y; M/F
8	Kalaskar et al. (2025) [33]	India	Roller skating	nRCT	Age range 8–14 y; M/F	Age range 8–14 y; M/F

**Table 4:** Characteristics of Group 1 and Group 2 in the included studies.

No.	Authors (Year)	Sample Size	Comparison (G1 vs. G2)	Type of Mouthguard (G1/G2)
1	McNutt et al. (1989) [26]	G1: 2167/G2: 303	MG users vs. non-users	G1: 60% stock, 30% boil-and-bite, 10% custom; G2: no MG.
2	LaBella et al. (2002) [27]	G1: 8663/G2: 62,273	MG users vs. non-users	G1: custom-fitted MG; G2: no MG.
3	Barbic et al. (2005) [29]	G1: 306/G2: 308	Specialised MG vs. standard MG	G1: WIPSS Brain-Pad; G2: standard MG of choice.
4	Finch et al. (2005) [30]	G1: 190/G2: 111	Custom MG (EVA) vs. mixed MGs	G1: custom-made (trilaminate EVA); G2: boil-and-bite and custom-made.
5	Lieger & von Arx (2006) [31]	G1: 43/G2: 224	MG users vs. non-users	G1: predominantly custom-made MG (39/43) and stock(4/43); G2: no MG.
6	Tanaka et al. (2015) [28]	G1: 69/G2: 431	High-school players vs. medical students	G1: standardised custom-made (EVA); G2: athlete’s choice (85.2% custom).
7	Singarapu et al. (2023) [32]	G1: 60/G2: 26	MG users vs. non-users	G1: ready-made (pre-formed); G2: no MG.
8	Kalaskar et al. (2025) [33]	G1: 43/G2: 43	MG users vs. non-users	G1: custom-made (EVA); G2: no MG.

**Table 5:** Type of mouthguard used in the included studies.

No.	Authors (Year)	MG Type (Study)	Material (Study)	Thickness (Study)	MG Type (Control)	Material (Control)	Thickness (Control)
1	McNutt et al. (1989) [26]	Mouthguard (type NS)	NR	NR	No mouthguard	NA	NA
2	LaBella et al. (2002) [27]	Custom-fitted MG	NR	NR	No mouthguard	NA	NA
3	Barbic et al. (2005) [29]	WIPSS Brain-Pad (boil-and-bite, double-layer)	NR	NR	Mixed: custom/boil-and-bite/no MG	NR	NR
4	Finch et al. (2005) [30]	Custom-fitted MG	EVA (trilaminate)	NR	Mixed: custom/boil-and-bite/no MG	NR	NR
5	Lieger & von Arx (2006) [31]	Mouthguard (type NS)	NR	NR	No mouthguard	NA	NA
6	Tanaka et al. (2015) [28]	Custom-made MG	EVA	4 mm	Athlete’s choice (mostly custom/OTC)	NR	NR
7	Singarapu et al. (2023) [32]	Custom-made MG	NR	NR	No mouthguard	NA	NA
8	Kalaskar et al. (2025) [33]	Custom-fitted MG	EVA	4 mm	No mouthguard	NA	NA

**Note:** For the purposes of this review, the terms “custom-made”, “custom-fitted”, and “custom” were considered equivalent and refer to mouthguards individually fabricated for a specific athlete. The terminology used in the table reflects that reported in the original studies. NR: not reported; NS: not specified; NA: not applicable; OTC: over-the-counter; MG: mouthguard; EVA: ethylene-vinyl acetate.

**Table 6:** Outcomes related to mouthguard use for the prevention of orofacial trauma.

No.	Authors (Year)	Intraoral Soft Tissue	Extraoral Soft Tissue	Dental Trauma	Bone Injuries	TMJ Injuries
1	McNutt et al. (1989) [26]	Reduction with MG * (SS, *p* = 0.0001)	NR	Reduction with MG * (SS, *p* = 0.0001)	Reduction with MG * (reported, NS)	Reduction with MG * (reported, NS)
2	LaBella et al. (2002) [27]	Reduction with custom-fitted MG (reported, NS)	NR	Reduction with custom-fitted MG (SS, *p* < 0.05)	NR	NR
3	Barbic et al. (2005) [29]	NR	NR	NR	NR	NR
4	Finch et al. (2005) [30]	NR	Reduction with custom-fitted MG (reported)	Reduction with custom-fitted MG (reported)	NR	NR
5	Lieger & von Arx (2006) [31]	Reduction with MG * (reported)	Reduction with MG * (reported)	Reduction with MG * (reported)	Reduction with MG * (reported)	NR
6	Tanaka et al. (2015) [28]	NR	NR	NR	NR	NR
7	Singarapu et al. (2023) [32]	Reduction with custom-made MG (reported)	NR	Reduction with custom-made MG (reported)	NR	Reduction with custom-made MG (SS, exact *p*-value not reported))
8	Kalaskar et al. (2025) [33]	Reduction with custom-fitted MG (SS, *p* = 0.002)	NS	Reduction with MG * (reported, NS)	Reduction with MG * (reported, NS)	Reduction with custom-fitted MG (SS, *p* < 0.001)

NR: not reported; NS: not significant (*p* > 0.05); SS: statistically significant (*p* < 0.05); Reported: descriptive clinical evidence without inferential statistical analysis; *: type of mouthguard not specified in the study; MG: mouthguard; TMJ: temporomandibular joint.

## Data Availability

The data presented in this study are available in the article.

## Abbreviations

The following abbreviations are used in this manuscript:

PRISMA-ScR	Preferred Reporting Items for Systematic reviews and Meta-Analyses extension for Scoping Reviews
PICOS	Population, Intervention, Comparison, Outcome, Study design
TDI	Traumatic dental injury
IADT	International Association of Dental Traumatology
OSF	Open Science Framework
TMJ	Temporomandibular joint
EVA	ethylene-vinyl acetate
USA	United States of America
Y	Year
M	Male
F	Female
NR	Not reported
RCT	Randomised controlled trial
nRCT	Non-randomised controlled trials
G1	Group 1
G2	Group 2
MG	Mouthguard
NS	Not specified
NA	Not applicable
OTC	Over-the-counter
SS	Statistically significant
